# Regulatory effects of berberine on intestinal microecology in mice with ulcerative colitis

**DOI:** 10.3389/fmicb.2025.1649947

**Published:** 2025-11-12

**Authors:** Xinyi Xu, Bin Zhao, Pingyu Liu, Xiaohui Tang, Zonglang Lai, Na Song, Jun Cheng

**Affiliations:** 1 Graduate School of Shanghai Medical College, Fudan University, Shanghai, China; 2 Chongqing Shapingba Hospital of Traditional Chinese Medicine, Chongqing, China; 3 Institute of Literature in Chinese Medicine, Nanjing University of Chinese Medicine, Nanjing, China; 4 Department of Oncology, Chongqing Hospital of Traditional Chinese Medicine, Chongqing, China

**Keywords:** berberine, ulcerative colitis, gut microbiota, metabolites, Mendelian randomization

## Abstract

**Background:**

Currently, therapeutic approaches for ulcerative colitis (UC), such as aminosalicylates, glucocorticoids, and biologics, exhibit certain efficacy but are hindered by limitations including side effects, high costs, or suboptimal responses. As a traditional Chinese medicine component, berberine (BBR) possesses anti-inflammatory properties and the ability to modulate the gut microbiota, with low toxicity, and may provide new directions for UC treatment in the future.

**Methods:**

A mouse UC model was established via dextran sulfate sodium (DSS) induction, and dose-and time-dependent screening was performed to determine the optimal BBR dosage and intervention duration for subsequent experiments. The disease activity index (DAI) and colon length were measured. Colonic tissue changes were observed via HE staining. Serum cytokine levels (IL-1*β*, TNF-*α*, IL-10, TGF-*β*) were detected using ELISA. The expression levels of ZO-1 and Occludin in mouse colonic tissues were detected by WB. Further analyses included 16S rRNA sequencing to assess gut microbiota diversity and composition, untargeted metabolomics to identify differential metabolites in intestinal tissues, and Mendelian randomization (MR) analysis to explore causal associations among intestinal genes, circulating metabolites, and key bacterial genera. Finally, functional validation was performed by inhibiting the PDGFA receptor.

**Results:**

Berberine significantly alleviated the DAI score, colonic pathological damage, and cytokine imbalance in UC mice, as well as restored mucosal barrier integrity, with the most pronounced effects observed in the UC + low-BBR 14 days group. Gut microbiota analysis revealed distinct microbial structures across groups, with the UC + low-BBR 14 days group showing significantly higher relative abundances of *Bacteroides*, *Alistipes*, and *unclassified_Clostridia_vadinBB60_group* compared to the UC group (*p* < 0.05). Metabolomics analysis indicated that berberine altered the composition of intestinal tissue metabolites and metabolic pathways. MR analysis demonstrated inverse causal associations between PDGFA and lithocholate sulfate, as well as between lithocholate sulfate and *Alistipes*. Additionally, inhibition of the PDGFA receptor reversed the therapeutic effects of BBR, exacerbating inflammatory responses and intestinal mucosal barrier damage. Finally, the correlation analysis between gut microbiota and metabolites also confirmed that the abundance of the genus *Alistipes* exhibited a highly significant negative correlation with lithocholate sulfate levels (*p* < 0.001).

**Conclusion:**

Berberine ameliorates symptoms of UC in mice by regulating gut microbiota and metabolite composition. MR analysis first establishes a unidirectional causal chain of PDGFA/lithocholate sulfate/*Alistipes*, and animal experiments confirm that blocking the PDGFA receptor reverses its therapeutic effects and aggravates inflammation and intestinal mucosal injury.

## Graphical abstract

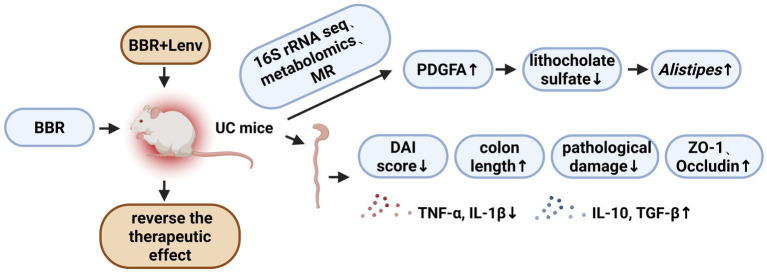

## Introduction

Ulcerative colitis (UC) is a chronic nonspecific inflammatory bowel disease characterized by continuous inflammation starting from the rectum and spreading proximally to the colon, involving the rectum and colon to varying degrees. It primarily affects the mucosal and submucosal layers of the intestine, severely impacting patients’ quality of life (Kobayashi et al., 2020; Li et al., 2022). Traditional treatments mainly include pharmacotherapy (e.g., aminosalicylates, glucocorticoids, immunosuppressants) and surgery. However, clinical challenges persist, such as steroid dependence, drug adverse reactions, and resistance to traditional medications in some patients. Therefore, developing safe and effective novel therapies remains an urgent challenge (Bressler et al., 2015).

Although genetic susceptibility (e.g., NOD2, IL23R mutations) (Okazaki et al., 2008) and immune dysregulation (excessive activation of Th1/Th17 cells) (Zhao et al., 2024) are core pathogenic factors of UC, an increasing number of studies have shown that the interactive disruption between gut microbiota dysregulation and host metabolic homeostasis is a key driver of disease progression. For instance, UC patients exhibit enrichment of pro-inflammatory bacterial genera and depletion of anti-inflammatory genera, accompanied by abnormal bile acid metabolism and insufficient short-chain fatty acid production (Zou et al., 2021). However, traditional observational studies struggle to clarify the causal direction among intestinal genes, metabolites, and microbiota. Mendelian randomization (MR), by using genetic variants associated with gene expression (e.g., expression quantitative trait loci, eQTL) as instrumental variables, can effectively circumvent environmental confounding biases and infer causal effects (Greenland, 2018).

Berberine, an isoquinoline alkaloid with multiple biological activities, has been widely confirmed to exhibit antibacterial, anti-inflammatory, and metabolic-regulatory effects (Zhu et al., 2022). In the context of UC, it exhibits several advantages over traditional therapies: it has low toxicity and minimal systemic exposure risk, while also avoiding the high costs and infection risks associated with injectable biologics. Notably, it demonstrates unique potential in regulating gut microecological balance. Previous studies have shown that berberine can improve microbiota dysregulation by enriching beneficial genera (such as *Bacteroides* and lactic acid-producing bacteria) and inhibiting the overproliferation of conditional pathogens, while also repairing the intestinal barrier through targeted regulation of tryptophan metabolism and the Wnt/*β*-catenin pathway to alleviate intestinal inflammation (Yang et al., 2023). However, the specific mechanisms underlying berberine’s effects in UC still require further in-depth investigation. This study aims to investigate the therapeutic effects of berberine on UC mice and its underlying mechanisms. Through 16S rRNA sequencing, untargeted metabolomics, and MR analysis, we will analyze the effects of berberine on gut microecology and metabolites, as well as the causal relationships between them, to provide a scientific basis for its application in UC treatment.

## Materials and methods

### Construction of ulcerative colitis mouse model

Forty-eight 8-week-old SPF-grade healthy male BALB/c mice, weighing 18 ~ 20 g, were provided by Chongqing Enswell Biotechnology Co., Ltd. All mice were acclimated to the environment under the same conditions for 7 days, then randomly divided into 6 groups using a computer-generated random number table (*n* = 8 per group) to ensure that there was no systematic bias in initial body weight and health status among the treatment groups. This study was conducted in strict accordance with the experimental protocols and ethical standards approved by the Institutional Animal Care and Use Committee. The administration dose of berberine was designed with reference to the widely reported and validated effective range in previous animal studies (Zhang et al., 2017; Hu et al., 2022).

Model induction and treatment: (1) Control group: No intervention; mice received a standard diet and water ad libitum. (2) UC group: Mice were fed a high-sugar/high-fat diet for 10 days, transferred to a custom-built 30 °C temperature chamber for 3 days, and finally administered 3% dextran sulfate sodium (DSS) in drinking water ad libitum for 7 days. (3) UC + low-BBR 7 days group: Following successful model induction, mice were orally gavaged with 0.2 mL of berberine (B875003, Macklin, China) solution at 100 mg/kg daily for 7 days. (4) UC + low-BBR 14 days group: Identical to Group 3, except the intervention was extended to 14 days. (5) UC + high-BBR 7 days group: Following model induction, mice received 0.2 mL of berberine solution at 300 mg/kg daily for 7 days. (6) UC + high-BBR 14 days group: Identical to Group 5, except the intervention was extended to 14 days. Upon completion of the intervention period, mice were anesthetized via intraperitoneal injection of 40 mg/kg pentobarbital sodium (P3761, BSZH, China). Blood was collected via orbital sinus puncture for serum separation. Euthanasia was performed by intravenous injection of 150 mg/kg pentobarbital sodium via the tail vein. Colonic tissues and fecal samples were harvested for subsequent analyses.

### Disease activity index (DAI) in mice

From the day of administration, mice were daily observed and recorded for mental status, food intake, water consumption, and activity. Body weight was measured at fixed intervals, and fecal consistency and stool bleeding were observed to calculate the DAI. The DAI was calculated as (body weight loss rate score + stool consistency score + intestinal bleeding severity score)/3, with the scoring criteria shown in Table 1.

**Table 1:** DAI scoring criteria.

Score	Body weight loss (%)	Stool consistency	Intestinal bleeding severity
0	0	Normal	Occult blood (−)
1	1 ~ 5	Soft but formed	Occult blood (−)
2	6 ~ 10	Soft and unformed	Occult blood (+)
3	11 ~ 18	Soft and moist	Visible blood in feces
4	>18	Watery stool	Massive rectal bleeding

### HE staining

Colon tissue paraffin sections from mice were dewaxed, cleared, and rehydrated. First, the sections were immersed in hematoxylin staining solution (G1004, Servicebio, China) for 5 min and then rinsed with tap water. Subsequently, the sections were stained in eosin staining solution (G1002, Servicebio, China) for 2 min and washed with distilled water. Finally, the sections underwent dehydration and coverslipping, followed by observation and imaging using an optical microscope (MF53, Mshot, China).

### ELISA

The expression levels of serum cytokines in mice were detected using mouse interleukin-1*β* (IL-1*β*) ELISA kits (CB10173-Mu, Coibo, China), mouse interleukin-10 (IL-10) ELISA kits (CB10161-Mu, Coibo, China), mouse tumor necrosis factor (TNF-*α*) ELISA kits (CB10851-Mu, Coibo, China), and mouse transforming growth factor (TGF-*β*) ELISA kits (CB10860-Mu, Coibo, China), according to the manufacturers’ instructions.

### Microbiota analysis

Total DNA was extracted from fecal samples, and PCR amplification was performed targeting the V3–V4 region of the 16S rRNA gene using forward primer (ACTCCTACGGGAGGCAGCA) and reverse primer (GGACTACHVGGGTWTCTAAT). Qualified PCR products were purified, quantified, and normalized to generate the final sequencing library. Raw sequencing reads were quality-controlled using the DADA2 method, including low-quality filtering with Trimmomatic v0.33 and primer identification and removal with cutadapt 1.9.1 to obtain high-quality clean reads. Nine samples yielded a total of 1,224,341 pairs of raw reads. After quality control and assembly, 1,150,183 clean reads were generated. The sequencing depth of each sample was no less than 74,914 reads, with an average depth of 127,798 reads. Using USEARCH (version 10.0), sequences were clustered at a 97% similarity level, and OTUs were filtered with a threshold of 0.005% of the total sequencing reads. Coverage indices were calculated to evaluate coverage, and the Coverage indices of all samples were higher than 0.999. Species annotation of OTUs was performed using QIIME2 software and the Silva 138 database, and community structure diagrams at various taxonomic levels were plotted using R tools. Shannon and Simpson indices were calculated via QIIME2, and significant differences were verified by Student’s *t*-test. Binary Jaccard distances were then calculated using QIIME2, and principal coordinate analysis (PCoA) plots were generated using R software.

### Untargeted metabolomics

Fifty milligrams of intestinal tissue samples were weighed and added to 1 mL of extraction solution containing internal standards (methanol: acetonitrile: water = 2:2:1), followed by vortex mixing. The samples were processed with a 45 Hz tissue grinder for 10 min and sonicated in an ice-water bath for 10 min. Subsequently, the samples were kept at −20 °C for 1 h, centrifuged at 12,000 rpm for 15 min at 4 °C, and 500 μL of the supernatant was transferred to EP tubes. The extracts were dried using a vacuum concentrator, reconstituted with 160 μL of rehydration solution (acetonitrile: water = 1:1), and vortexed. After sonication in an ice-water bath for 10 min and centrifugation at 12,000 rpm at 4 °C, 120 μL of the supernatant was transferred to 2 mL injection vials. Ten microliters from each sample were combined to form a quality control (QC) sample for instrument analysis.

The liquid chromatography-mass spectrometry (LC–MS) system for metabolomics analysis consisted of a Waters Acquity I-Class PLUS ultra-performance liquid chromatography (UPLC) system coupled with a Waters Xevo G2-XS QTOF high-resolution mass spectrometer (HRMS), using a Waters Acquity UPLC HSS T3 column (1.8 μm, 2.1 × 100 mm). The Xevo G2-XS QTof HRMS was operated under MassLynx V4.2 software in MSe mode for acquiring primary and secondary mass spectrometry data. The ESI ion source parameters were set as follows: capillary voltage at 2500 V (positive ion mode) and −2000 V (negative ion mode), cone voltage at 30 V, ion source temperature at 100 °C, desolvation gas temperature at 500 °C, backflush gas flow rate at 50 L/h, desolvation gas flow rate at 800 L/h, and mass range at 50 ~ 1,200 m/z. Raw data were processed for peak extraction and alignment using Progenesis QI software, and metabolites were identified by matching with the online METLIN database, public databases, and the BMK in-house library. Unit Variance Scaling (UV) was used for data normalization. To monitor data quality and the stability of the analysis process, quality control (QC) samples prepared by mixing extracts from all samples were used, and were injected periodically throughout the entire analysis process.

Principal component analysis (PCA) and orthogonal partial least squares discriminant analysis (OPLS-DA) were performed to analyze sample distribution and intergroup differences. Differential metabolites were screened based on variable importance in projection (VIP) from OPLS-DA models and combined with *p*-values from univariate analysis, with criteria set as VIP ≥ 1 and *p* < 0.05. KEGG pathway enrichment analysis of differential metabolites was conducted using the hypergeometric test in the clusterProfiler package. All statistical analyses were performed using R software (version 3.6.1) and related packages.

### Mendelian randomization

Exposure factors were defined as gene eQTLs in colon tissue from the GTEx V10 dataset. Lead SNPs for each gene (single SNP with the most significant *p*-value and *p* < 5e-8) were screened. Mediator factors included GWAS data for 1,400 circulating metabolites and their proportions (GCST90199621–GCST90201020), with a *p* < 1e-5 threshold set to avoid data loss due to overly strict filtering. Outcome factors were GWAS data for 473 gut microbiota species intersecting with the dominant microbiota in the treatment group (*Alistipes*; GCST90032186), with rows containing empty SNPs excluded. Local linkage disequilibrium pruning was performed using the ieugwasr package (reference: 1000 Genomes data; parameters: clump_kb = 10,000, clump_r2 = 0.001, clump_p = 1). For MR analysis, the Inverse Variance Weighted (IVW) method was used when the number of SNPs >1, and the Wald ratio method was applied when SNPs = 1. Directionality tests were performed for significant results (retaining significant test outcomes). Heterogeneity and horizontal pleiotropy tests were conducted for datasets with SNPs >1. Combined with differential metabolite data, complete causal chains were constructed, and genes corresponding to target eQTLs were selected as complete mediators.

### Molecular docking

The structures of the ligand lithocholate sulfate (PubChem CID: 122391354) and receptor PDGFA (PDB ID: 3MJK) were constructed using OpenBabel 3.1.1 and PyMOL 3.1.0, respectively. Following hydrogenation and charge optimization of the receptor, as well as conformational searches of the ligand, performed via AutoDockTools 1.5.7, molecular docking was carried out using AutoDock Vina 1.2.7. Docking complexes were analyzed for interactions using PLIP and visualized in PyMOL.

### Intervention of low-dose berberine combined with Lenvatinib in UC mice

Thirty-two 8-week-old SPF-grade healthy male BALB/c mice (body weight: 18 ~ 20 g) were provided by Chongqing Enswell Biotechnology Co., Ltd. All mice were acclimated under identical conditions for 7 days and then randomly divided into four groups (8 mice per group).

Grouping and treatment: (1) Control group: No intervention; mice received a standard diet and water ad libitum. (2) UC group: Maintained on a normal diet and water after UC modeling. (3) BBR treatment group: After successful modeling, mice were orally gavaged with 0.2 mL of BBR solution (100 mg/kg) daily for 14 days. (4) BBR treatment + Lenvatinib group: After successful modeling, mice received BBR gavage at the above dose and duration, and were intraperitoneally injected with 10 mg/kg Lenvatinib (HY-10981, MCE, United States) 3 days before euthanasia. At the end of the intervention, orbital blood and colonic tissues were collected for subsequent analyses, and colon length was recorded.

### WB

Samples were lysed to extract total protein using protein lysis buffer (P0013B, Beyotime, China) supplemented with protease and phosphatase inhibitors (P1045, Beyotime, China). The extracted total proteins were separated by SDS-polyacrylamide gel electrophoresis (SDS-PAGE) and then transferred to PVDF membranes (10,600,023, Amersham, Germany). After blocking for 1 h, the membranes were washed and incubated with primary antibodies at 4 °C overnight. The next day, the membranes were incubated with secondary antibodies at room temperature for 60 min, followed by developing protein bands using an ECL enhanced chemiluminescence detection kit (34,580, Thermo, United States) and detection with a gel imaging system (Universal Hood II, Bio-Rad, United States). Band gray values were analyzed using ImageJ software, and relative protein expression levels were normalized with GAPDH as the internal reference.

### Data statistics

Data were analyzed and plotted using GraphPad Prism 9.0.0 software. One-way analysis of variance (one-way ANOVA) was used to assess differences between groups, with Tukey’s test performed for post-hoc multiple comparisons. Data were confirmed to have no outliers by Grubbs test, and all data were included in the analysis. Results were presented as “mean ± standard deviation (SD),” and *p* < 0.05 were considered statistically significant.

## Results

### Effects of berberine on ulcerative colitis mice

**Figure 1:**
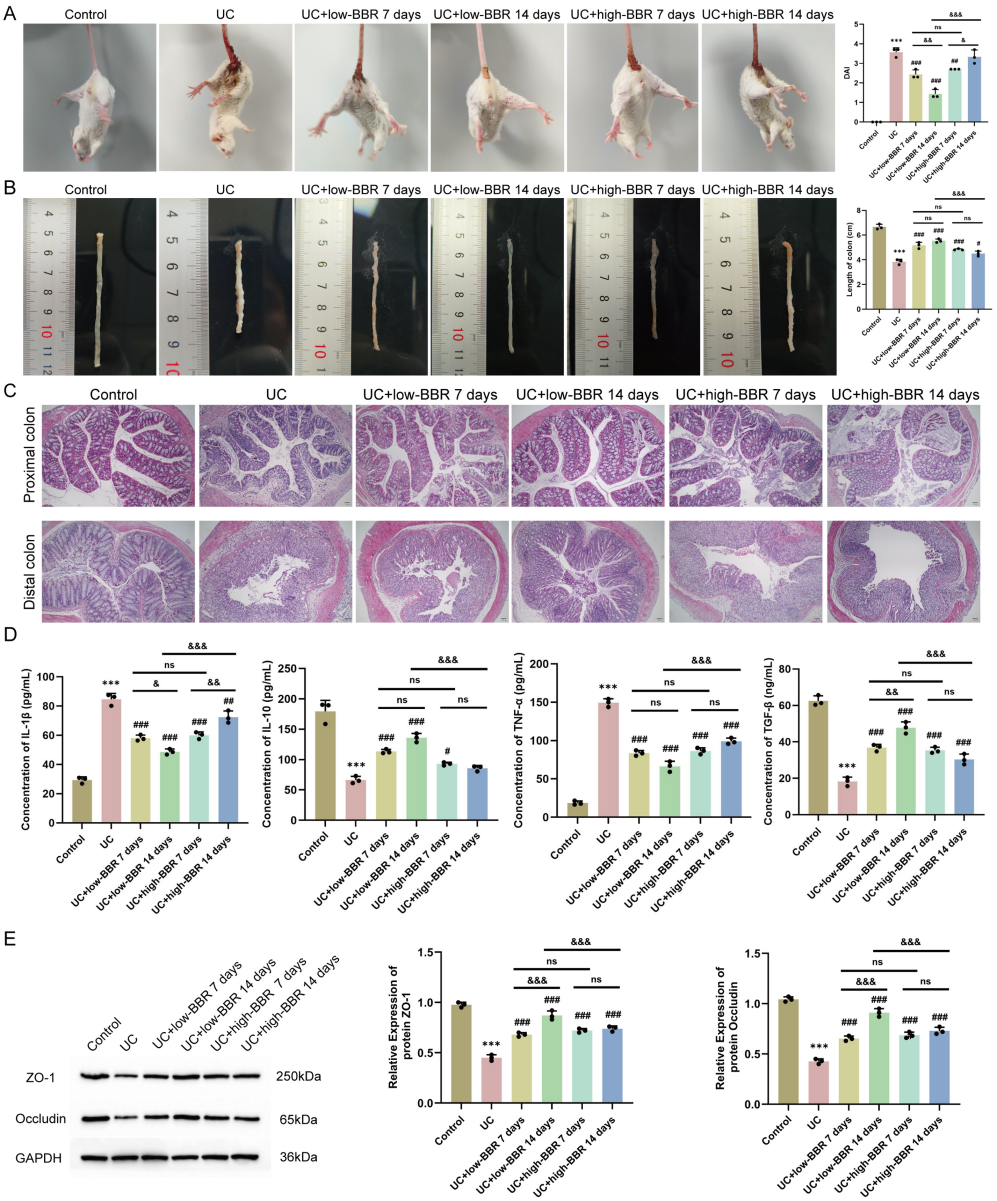
Effects of berberine on disease activity, tissue pathology, and serum cytokines in ulcerative colitis mice. **(A)** DAI scores. **(B)** Colon length. **(C)** Pathological changes in the proximal and distal colon observed by HE staining. **(D)** Expression levels of serum IL-1*β*, IL-10, TNF-*α*, and TGF-*β* in mice detected by ELISA. **(E)** WB detection of protein expression of ZO-1 and Occludin in mouse colonic tissues. Compared with the control group, *** *p* < 0.001; compared with the UC group, ^#^ *p* < 0.05, ^##^ *p* < 0.01, ^###^ *p* < 0.001; intergroup comparisons, ^&^ *p* < 0.05, ^&&^ *p* < 0.01, ^&&&^ *p* < 0.001. Scale bar = 50 μm.

We followed the method described by Huang et al. (2025) and confirmed successful model establishment through DAI score assessment, colon length measurement, and HE staining (Figure 1). To investigate the effects of BBR on UC, we used low-dose and high-dose BBR to orally gavage UC mice. To determine whether there was a correlation between drug treatment and treatment duration, we collected tissue and peripheral blood samples on days 7 and 14 for analysis. Compared with the UC group, the UC + low-BBR 7 days, UC + low-BBR 14 days, and UC + high-BBR 7 days groups all had significantly lower DAI scores (*p* < 0.01, Figure 1A), and all treatment groups had significantly extended colon lengths (*p* < 0.05, Figure 1B). In terms of pathological damage, the UC + low-BBR 7 days group showed reduced proximal colon crypt atrophy and alleviated inflammation associated with goblet cell reduction; although distal gland loss and epithelial detachment persisted, the intestinal lumen bleeding area was smaller, and the number of infiltrating inflammatory cells was reduced. The UC + low-BBR 14 days group exhibited nearly normal proximal colon tissue structure, with only mild epithelial damage, neatly arranged distal glands, punctate submucosal bleeding, and minimal inflammation, demonstrating the most pronounced improvement. The UC + high-BBR 7 days group and UC + high-BBR 14 days group showed slightly reduced proximal colon crypt atrophy and intestinal lumen bleeding, with modest improvement in distal goblet cell deformation, but significant tissue disorder and inflammation remained (Figure 1C). Additionally, compared with the UC group, all treatment groups had decreased serum IL-1*β* and TNF-*α* levels and increased serum IL-10 and TGF-*β* levels (Figure 1D). To further investigate the repair effect of BBR on intestinal barrier function, we detected the expression of ZO-1 and Occludin. Results showed that compared with the UC group, the protein expression levels of ZO-1 and Occludin in the colonic tissues of mice in all treatment groups were significantly increased (Figure 1E). These results indicate that berberine can effectively reduce the severity of UC.

Further analysis revealed that in the low-dose group, extending the intervention duration to 14 days could achieve better therapeutic effects. Specifically, this was manifested by a further decrease in the DAI score (*p* < 0.01, Figure 1A) and serum IL-1*β* level, while the TGF-*β* level (*p* < 0.05, Figure 1D) and the expression levels of the tight junction proteins ZO-1 and Occludin in colonic tissues were significantly increased (*p* < 0.05, Figure 1E). In contrast, in the high-BBR group, prolonged intervention trended toward increased DAI score and IL-1*β* levels (*p* < 0.05, Figures 1A,D). No significant differences were observed in any index between the UC + low-BBR 7 days group and UC + high-BBR 7 days group (*p* > 0.05, Figure 1). In summary, BBR can effectively alleviate disease activity, pathological damage, and cytokine imbalance in UC mice, as well as restore mucosal barrier integrity. In the model of this study, continuous intervention with a low dose for 14 days is the optimal therapeutic regimen.

### Effects of berberine on gut microbiota diversity and composition in ulcerative colitis mice

**Figure 2:**
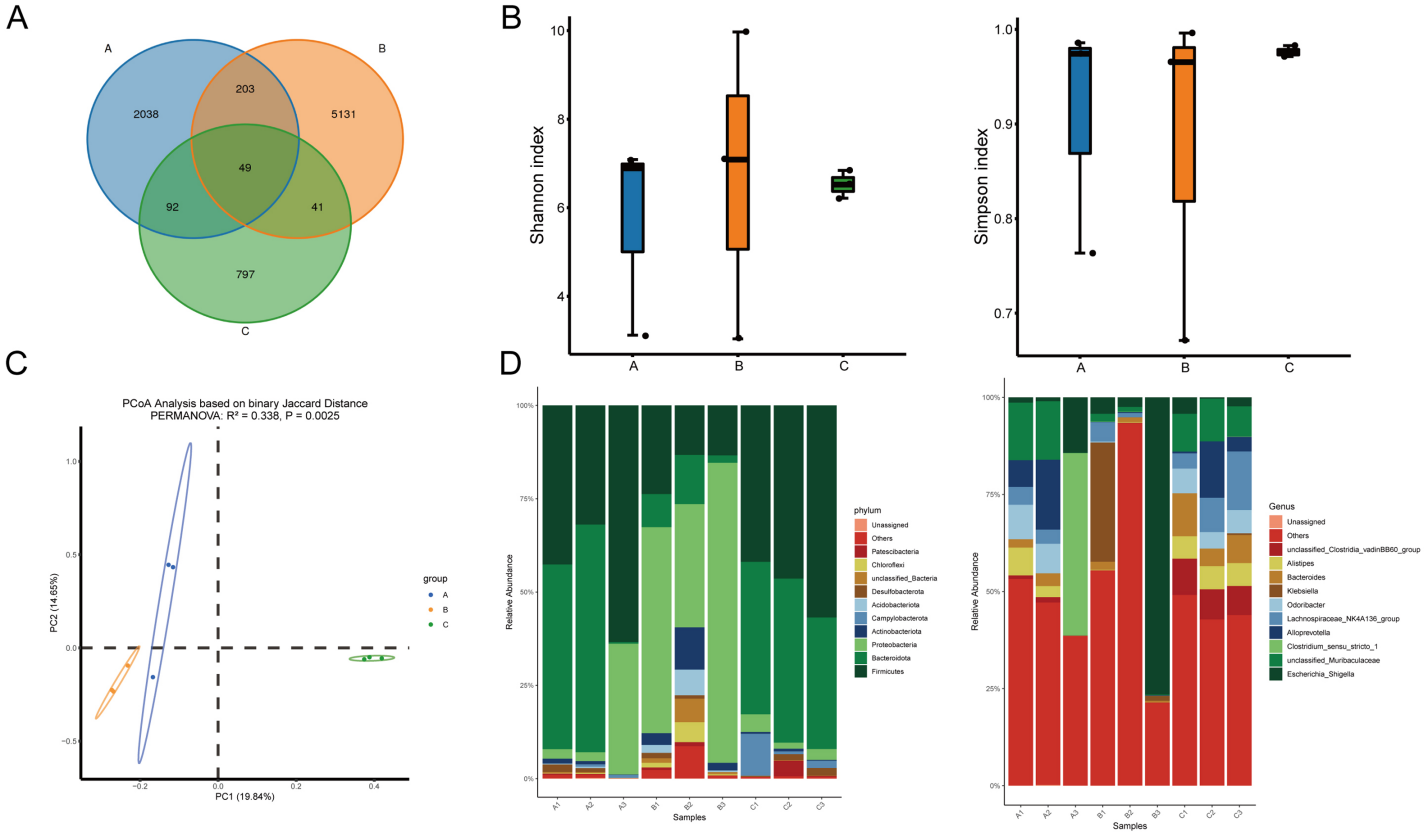
Differential analysis of gut microbiota composition. **(A)** Venn diagram. **(B)** Alpha diversity analysis. **(C)** PCoA analysis. **(D)** Composition of gut microbiota at the phylum and genus levels. A: Control group, B: UC group, C: UC + low-BBR 14 days group.

The above results indicated that the UC + low-BBR 14 days group exhibited the best therapeutic effect in ulcerative colitis mice. Based on this, this study further explored the regulatory effects of berberine on the gut microecology of UC mice by analyzing differences in gut microbiota composition among the control group, UC group, and UC + low-BBR 14 days group. A Venn diagram at the OTU level showed that the control group, UC group, and UC + low-BBR 14 days group had 2,382, 5,424, and 979 OTUs, respectively, with 49 OTUs shared among the three groups (Figure 2A). *α*-diversity analysis revealed no significant differences in Shannon and Simpson indices of gut microbiota among groups (Figure 2B), suggesting that berberine intervention did not significantly alter the richness or overall diversity of gut microbiota. PCoA based on Binary Jaccard distance explained 34.49% of the variable composition across the two principal components (Figure 2C). The microbial community structures of the control group, UC group, and UC + low-BBR 14 days group were significantly separated, indicating obvious differences in microbiota composition among the three groups. Furthermore, statistical evaluation of differences in the overall community structure was conducted via PERMANOVA, and the results confirmed that significant differences existed among groups (*p* = 0.002, R^2^ = 0.338). At the phylum level, the dominant phyla in all groups were primarily Firmicutes, Bacteroidota, and Proteobacteria. At the genus level, the dominant genera in all groups were mainly *Escherichia_Shigella*, *unclassified_Muribaculaceae*, and *Clostridium_sensu_stricto_1* (Figure 2D).

To gain insight into the specific differences in gut microbiota composition among groups, visual analysis was conducted on the top 10 bacterial genera at the genus level (Supplementary Figure S1). Results showed that compared with the UC group, the UC + low-BBR 14 days group had significantly higher relative abundances of *Bacteroides*, *Alistipes*, and *unclassified_Clostridia_vadinBB60_group* (*p* < 0.05). In contrast, there were no significant changes in the relative abundances of *Escherichia_Shigella*, *unclassified_Muribaculaceae*, *Clostridium_sensu_stricto_1*, *Alloprevotella*, *Lachnospiraceae_NK4A136_group*, *Odoribacter*, and *Klebsiella* among groups (*p* > 0.05). These results indicate that berberine had no significant effect on the overall richness and diversity of gut microbiota in UC mice but could reshape the gut microbial community structure by specifically regulating the relative abundances of certain genera.

### Effects of berberine on intestinal tissue metabolites in ulcerative colitis mice

**Figure 3:**
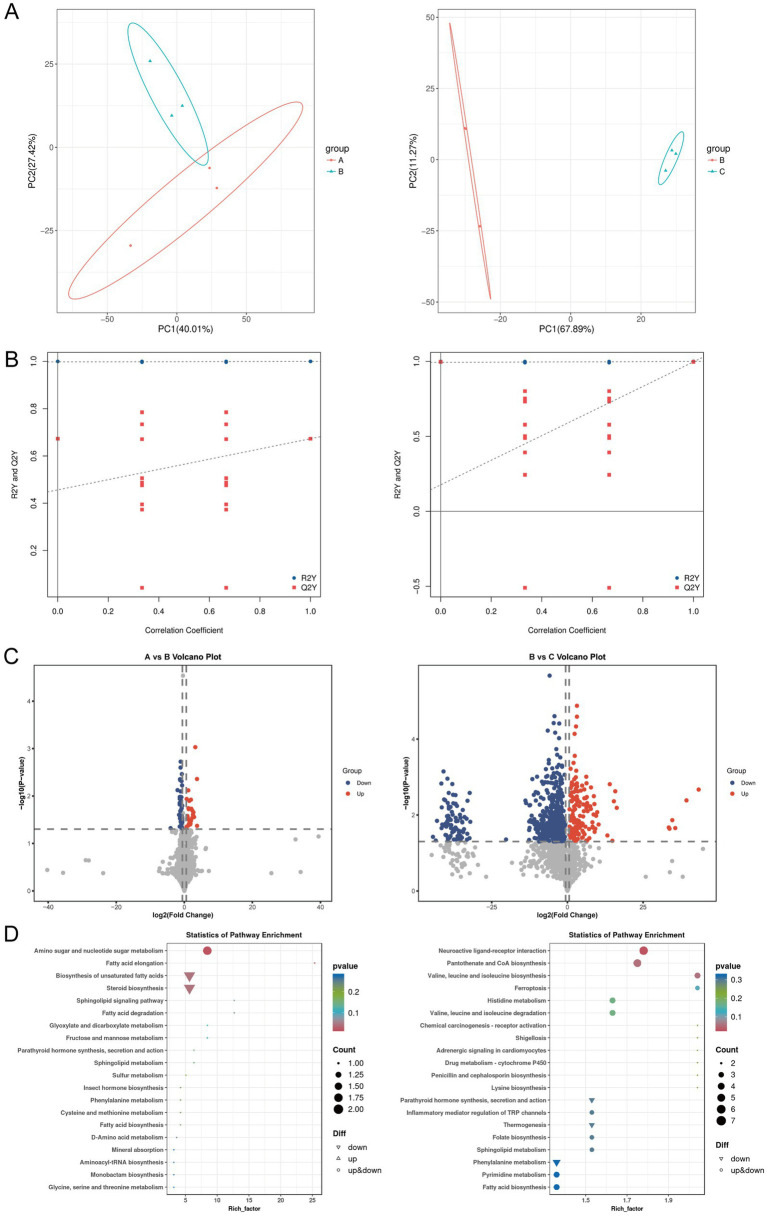
Differential analysis of intestinal tissue metabolites. **(A)** PCA results between groups A and B, and between groups B and C. **(B)** OPLS-DA model permutation test plots between groups A and B, and between groups B and C. **(C)** Volcano plots of differential metabolites between groups A and B, and between groups B and C. The abscissa represents the logarithmic fold change in metabolite concentration (Log₂ fold change), which denotes the effect size; the ordinate represents the negative logarithmic transformation value of statistical significance (−Log₁₀ *p*-value). The dashed lines mark the significance threshold (*p* < 0.05) and the fold change threshold (|Log₂ fold change| > 0.58). Each dot represents a metabolite, where red dots indicate significantly upregulated metabolites, blue dots indicate significantly downregulated metabolites, and gray dots indicate metabolites with no significant change. **(D)** KEGG enrichment results of differential metabolites between groups A and B, and between groups B and C. A: Control group, B: UC group, C: UC + low-BBR 14 days group.

Given that dynamic changes in gut microbiota can influence host physiological functions through metabolic pathways, this study further explored the potential mechanisms of berberine in improving ulcerative colitis using untargeted metabolomics to analyze metabolic differences and related pathways in intestinal tissues of mice across groups. PCA showed that samples from different groups exhibited characteristic distributions, with obvious separation observed between the control and UC groups, as well as between the UC and UC + low-BBR 14 days groups (Figure 3A). OPLS-DA permutation test plots demonstrated that the models for control vs. UC and UC vs. UC + low-BBR 14 days groups exhibited reliable intergroup discriminative power and data robustness (Figure 3B). Volcano plot analysis visually displayed the overall trends in metabolite content differences between groups. As shown in Figure 3C, 65 differential metabolites were identified between the control and UC groups (|logFC| > 0.58, *p* < 0.05), and 692 differential metabolites were detected between the UC and UC + low-BBR 14 days groups (|logFC| > 0.58, *p* < 0.05). Finally, KEGG enrichment analysis identified the main biological function pathways enriched by differential metabolites. As shown in Figure 3D, differential metabolites between the control and UC groups were primarily enriched in pathways such as Amino sugar and nucleotide sugar metabolism, Fatty acid elongation, and Biosynthesis of unsaturated fatty acids. Differential metabolites between the UC and UC + low-BBR 14 days groups were mainly enriched in Neuroactive ligand-receptor interaction, Pantothenate and CoA biosynthesis, and Valine, leucine and isoleucine biosynthesis pathways.

### Causal associations between intestinal genes, circulating metabolites, and *Alistipes* genus based on Mendelian randomization analysis

**Figure 4:**
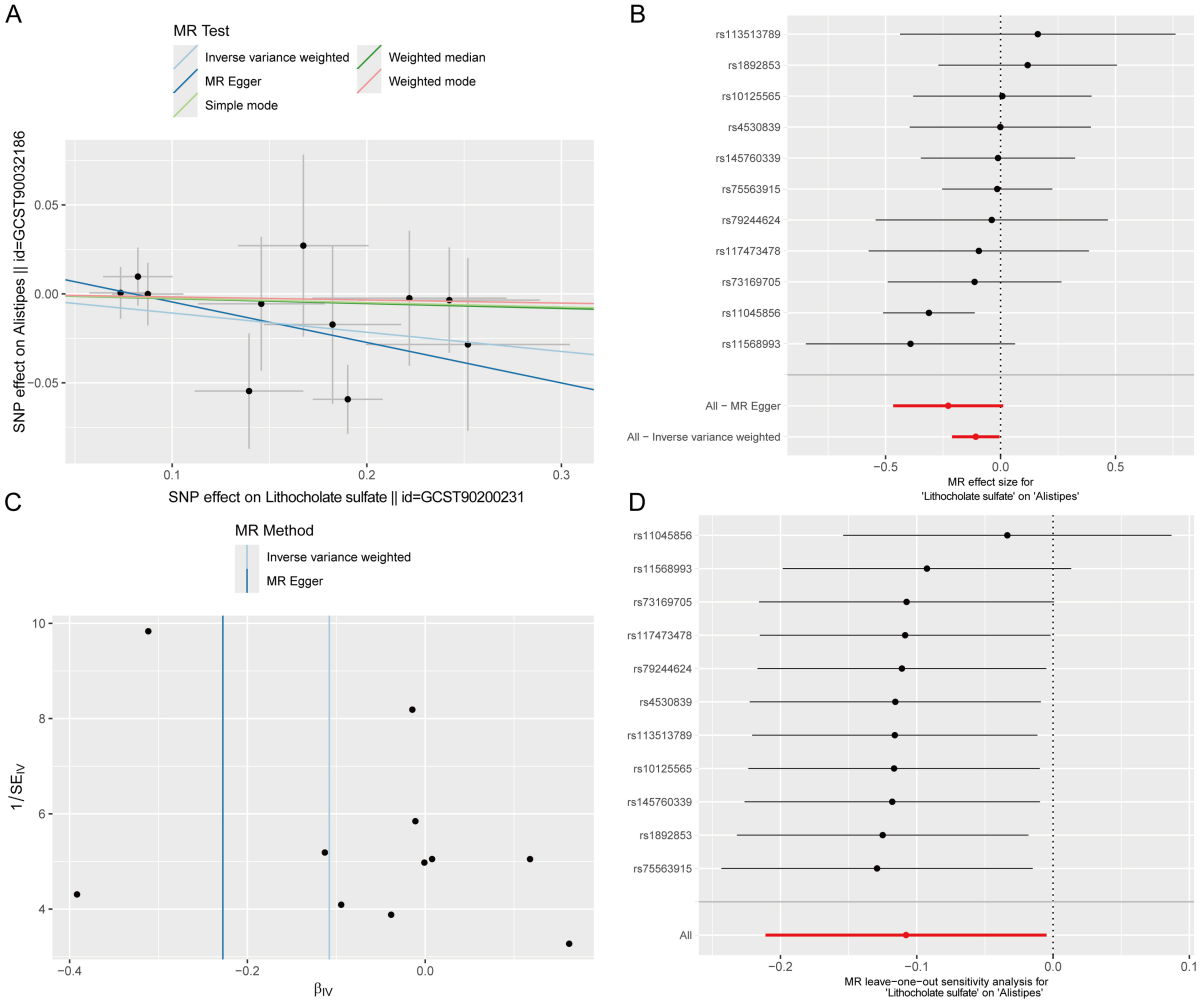
Causal effects of circulating metabolites on the abundance of the dominant gut microbiota *Alistipes*. **(A)** Scatter plot. **(B)** Forest plot. **(C)** Funnel plot. **(D)** Leave-one-out sensitivity analysis plot.

Given that gut microbiota metabolites can affect circulating metabolite levels through the gut-blood barrier, and MR can infer causal relationships based on genetic variants, with some studies directly using circulating metabolites as surrogate markers for gut microbiota metabolic activity (Wang et al., 2023; He et al., 2024; Zhao et al., 2025; Wang and Lv, 2024). This study included data of 1,400 circulating metabolites and explored the causal associations between dominant gut microbiota and metabolites via MR analysis. As reported in the literature, *Alistipes* could alleviate UC through multiple pathways such as repairing intestinal barrier and regulating inflammatory signals (Lin et al., 2025; Parker et al., 2020), so this genus was selected for Mendelian randomization analysis. MR results of 1,400 circulating metabolites and dominant microbiota *Alistipes* showed that 53 metabolites passed the test (Supplementary Figure S2), including 13 unnamed metabolites, 11 metabolite ratios and 29 known metabolites. Notably, a Venn diagram of 1,400 blood-circulating metabolites and differential metabolites in intestinal tissues showed that two metabolites (lithocholate sulfate and butyryl-L-carnitine) were shared among the three groups (Supplementary Figure S3). Among them, only lithocholate sulfate was simultaneously present in the differential metabolites of intestinal tissues and the causal relationships identified by MR (Supplementary Figures S2, S4), suggesting that this metabolite may act as a key mediator between the intestinal tract and blood circulation. The MR scatter plot of Lithocholate sulfate and *Alistipes* showed a slope less than 0, indicating that Lithocholate sulfate was a protective factor for *Alistipes* (Figure 4A). The forest plot showed that the elevation of Lithocholate sulfate level could reduce the abundance of *Alistipes* (Figure 4B). The funnel plot suggested that SNPs might have heterogeneity (Figure 4C). Sensitivity analysis showed that the error bars of effect values had no significant changes after removing individual SNPs, indicating the robustness of the results (Figure 4D). Heterogeneity and horizontal pleiotropy analyses are shown in Supplementary Table S1.

**Figure 5:**
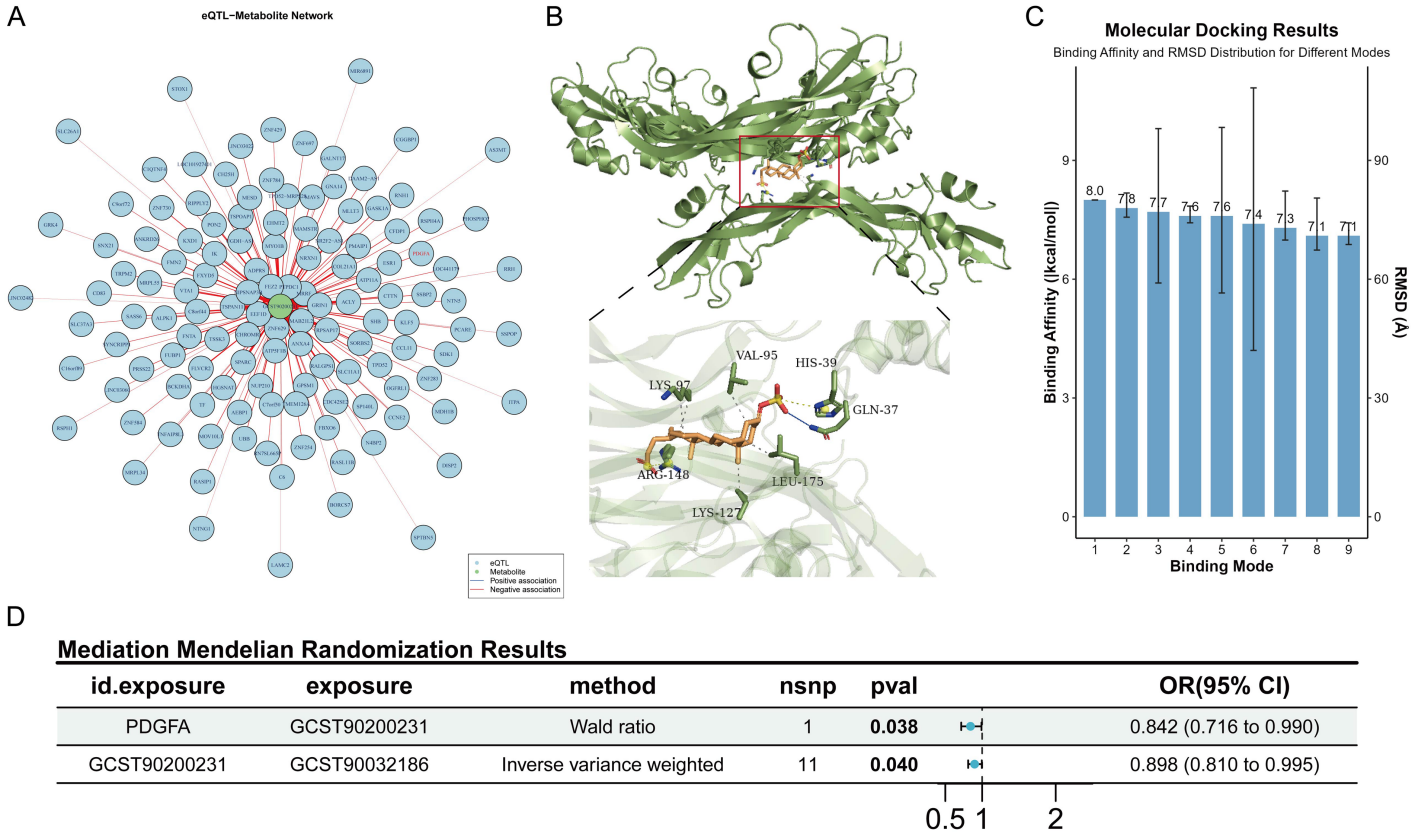
MR protective effects of intestinal genes on lithocholate sulfate. **(A)** MR association network diagram between intestinal genes and lithocholate sulfate. **(B)** Schematic diagram of molecular docking binding sites and key amino acid residues. **(C)** Distribution of binding affinities and RMSD values across different binding conformations. **(D)** Schematic of the complete mediation forest plot.

Further, using lead single-nucleotide polymorphisms (SNPs) of genes from intestinal expression quantitative trait loci (eQTLs) as instrumental variables, MR analysis was performed between 4,975 colon eQTL-corresponding genes (from the GTEx database, V10 version) and lithocholate sulfate. For each gene, only one lead SNP with *p* < 5 × 10^−8^ was selected. Results showed that 257 genes passed the test, among which 144 were inverse causals and 113 were positive causals. Previous studies have reported that the PDGF family promotes the proliferation of fibroblasts and astrocytes, participates in wound healing and tissue repair, and PDGF-stimulated angiogenesis significantly accelerates the healing of ulcerative colitis (UC) (Deng et al., 2013; Horikawa et al., 2015). Consistent with these findings, our results showed that PDGFA appeared in the inverse causal network (Figure 5A). Therefore, a molecular docking experiment was performed on PDGFA. The results showed that the best binding mode of Lithocholate sulfate and PDGFA had strong binding affinity (−8.0 kcal/mol), and the RMSD value between the optimal conformation and the suboptimal conformation was 2.347 Å. Multiple binding modes had similar affinities (−7.8 ~ −7.1 kcal/mol), indicating that the ligand had a stable dominant conformation in the binding pocket (Figures 5B,C). Mediation MR analysis confirmed that PDGFA was an inverse causal factor for Lithocholate sulfate level (b = −0.172, *p* = 0.038), and Lithocholate sulfate was an inverse causal factor for *Alistipes* (b = −0.108, *p* = 0.040, Figure 5D).

### Effects of PDGFA receptor inhibition on ulcerative colitis mice

**Figure 6:**
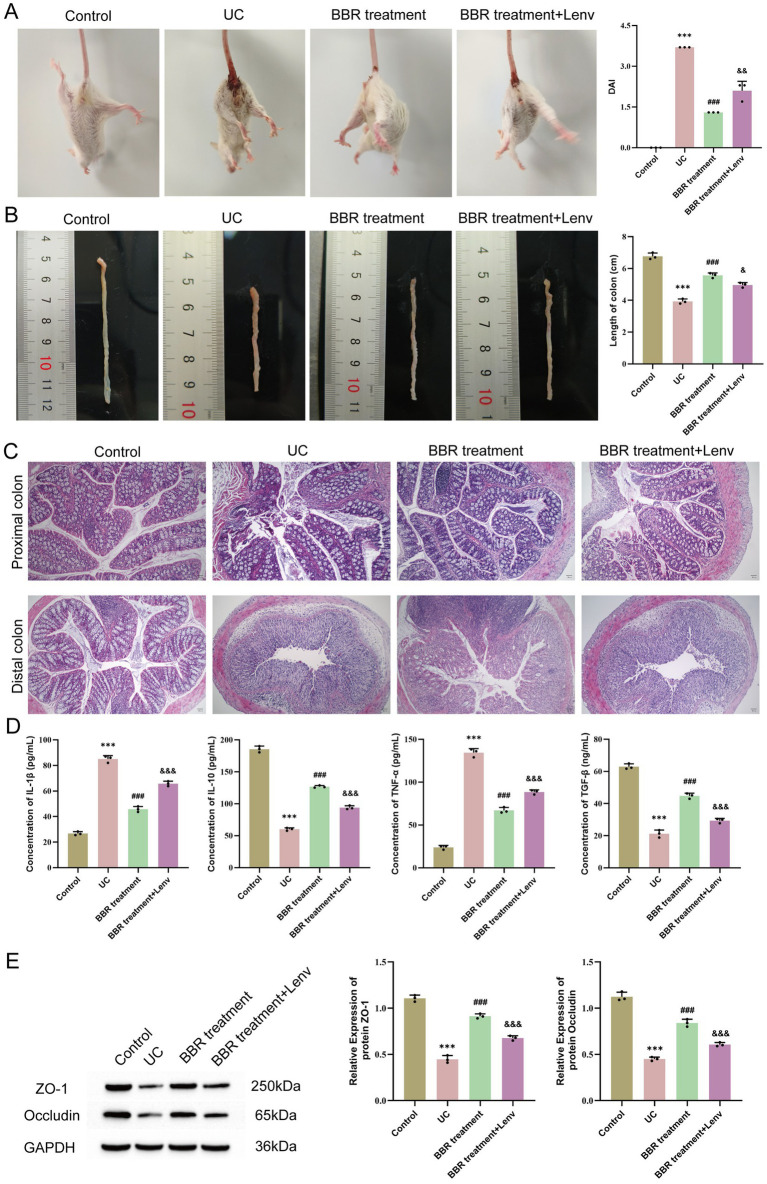
Effects of PDGFA on disease activity, tissue pathology, and serum cytokines in ulcerative colitis mice. **(A)** DAI scores. **(B)** Colon length. **(C)** Pathological changes in the proximal and distal colon observed by HE staining. **(D)** Expression levels of serum IL-1*β*, IL-10, TNF-*α*, and TGF-*β* detected by ELISA. **(E)** WB detection of protein expression of ZO-1 and Occludin in mouse colonic tissues. Compared with the control group, *** *p* < 0.001; compared with the UC group, ^##^ *p* < 0.01, ^###^ *p* < 0.001; compared with the UC + low-BBR 14 days group, ^&^ *p* < 0.05, ^&&^ *p* < 0.01, ^&&&^ *p* < 0.001. Scale bar = 50 μm.

Based on the causal conclusions from MR analysis, inverse causal associations were identified between PDGFA and lithocholate sulfate levels, as well as between lithocholate sulfate and the *Alistipes* genus. To further validate the biological function of PDGFA in UC, the PDGFA receptor inhibitor Lenvatinib was used to intervene in a UC mouse model. Compared with the BBR treatment group, the BBR treatment + Lenvatinib group had a significantly higher DAI score (*p* < 0.01, Figure 6A) and significantly shorter colon length (*p* < 0.05, Figure 6B). Histomorphological analysis revealed that the BBR treatment group exhibited nearly normal proximal colon tissue morphology with orderly arranged epithelial cells, only occasional crypt damage and deformation, and local distal colon mucosal epithelial damage, partial crypt loss, reduced goblet cells, and punctate submucosal bleeding. In the BBR treatment + Lenvatinib group, however, proximal colon showed epithelial cell rupture and lysis, crypt atrophy and shedding, and reduced goblet cells, while distal colon tissues were disorderly arranged, with nearly disappeared glandular structures, extensive epithelial shedding, and exacerbated inflammatory infiltration (Figure 6C). As shown in Figure 6D, ELISA detection of serum cytokine levels revealed that compared with the BBR treatment group, the BBR treatment + Lenvatinib group had significantly increased serum pro-inflammatory cytokines IL-1*β* and TNF-*α* (*p* < 0.001) and significantly decreased anti-inflammatory cytokines IL-10 and TGF-*β* (*p* < 0.001). Furthermore, WB results showed that the expression levels of ZO-1 and Occludin in the colonic tissues of the BBR treatment + Lenvatinib group were significantly lower than those in the BBR treatment group (*p* < 0.05, Figure 6E). In conclusion, inhibition of the PDGFA receptor disrupted the intestinal mucosal barrier and exacerbated the inflammatory response and tissue damage in UC mice.

### Correlation analysis between gut microbiota and intestinal tissue metabolites

**Figure 7:**
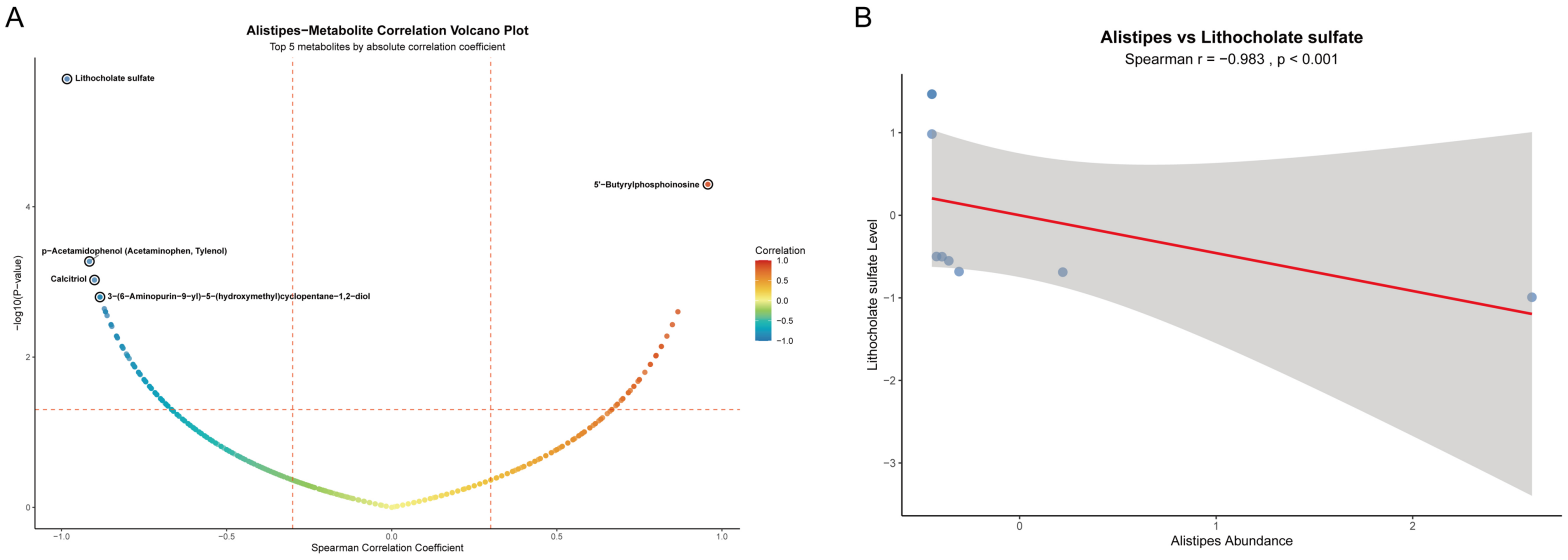
Correlation analysis between the genus *Alistipes* and intestinal metabolites. **(A)** Volcano plot of Spearman correlation between the genus *Alistipes* and all intestinal metabolites; **(B)** Scatter plot of the correlation between the relative abundance of the genus *Alistipes* and lithocholate sulfate level.

To further clarify the association characteristics between the genus *Alistipes* and all detected intestinal tissue metabolites, we performed Spearman correlation analysis (Figure 7A). Results showed that a variety of metabolites had significant correlations with the abundance of the genus *Alistipes*. The top 5 metabolites in terms of absolute correlation coefficient with the abundance of *Alistipes* were Lithocholate sulfate, 5’-Butyrylphosphoinosine, p-Acetamidophenol, Calcitriol, and 3-(6-Aminopurin-9-yl)-5-(hydroxymethyl) cyclopentane-1,2-diol. Lithocholate sulfate was the metabolite with the strongest negative correlation with the genus *Alistipes*. To further verify this key association, we specifically plotted a scatter plot of the relative abundance of the genus *Alistipes* versus the level of Lithocholate sulfate (Figure 7B). Results showed that there was a very significant strong negative correlation between them (*r* = −0.983, *p* < 0.001). This result is consistent with the conclusion of our aforementioned Mendelian randomization analysis, and together they strongly indicate that the decrease in Lithocholate sulfate level is closely associated with the enrichment of the genus *Alistipes*, suggesting that this metabolite may be a key factor mediating the regulation of this beneficial bacterium by berberine.

## Discussion

UC is a chronic intestinal inflammatory disease, and current therapies suffer from insufficient efficacy and side effects (AlAmeel et al., 2023). BBR, a natural alkaloid, exhibits potential in anti-inflammation and gut microbiota regulation, but its mechanism in treating UC remains unclear. This study found that berberine significantly improved disease activity, colonic pathological damage, and cytokine imbalance in UC mice, consistent with findings reported by Zhu et al. (2019). Additionally, the UC + low-BBR 14 days group showed the optimal therapeutic effect, while the UC + high-BBR 14 days group displayed poor efficacy and even exacerbated inflammatory responses. This may be attributed to the excessive inhibition of gut microbiota by high-dose BBR or metabolic burden on the host, suggesting that dose optimization is critical for clinical application.

Recent studies have indicated that gut microecological imbalance may be a key factor triggering and exacerbating ulcerative colitis (Yuan et al., 2021). In this study, although *α*-diversity of gut microbiota did not differ significantly among groups, microbial community structures showed obvious differences. Studies have shown that gut microbiota in UC mice is characterized by imbalanced proportions of Firmicutes and Proteobacteria (Yin et al., 2022). Within Proteobacteria, overproliferation of *Escherichia_Shigella* is closely associated with mucosal inflammation, and increased relative abundance of this genus in the gastrointestinal tract often induces diarrhea and inflammatory responses (Su, 2024). The results of this experiment were consistent with prior studies: the relative abundance of *Escherichia_Shigella* in the UC group was higher than that in the control group, and the UC + low-BBR 14 days group showed a tendency toward reduced abundance of this genus compared with the UC group, although the difference was not statistically significant, possibly due to the small sample size. *Bacteroides* plays important roles in nutrient absorption, anti-inflammation, and promotion of barrier function (Yan et al., 2023). *Alistipes*, as a short-chain fatty acid-producing bacterium, alleviates UC through anti-inflammatory effects (Duan et al., 2025), while the *Clostridia_vadinBB60_group* participates in bile acid metabolism and antagonizes pathogen colonization to reduce inflammation. This study found that the relative abundances of *Bacteroides*, *Alistipes*, and *unclassified_Clostridia_vadinBB60_group* were significantly higher in the UC + low-BBR 14 days group than in the UC group. These results indicate that berberine improves gut health in UC mice by increasing the relative abundances of beneficial bacteria and decreasing those of harmful bacteria. Furthermore, untargeted metabolomics analysis of intestinal tissue metabolites revealed significant differences in metabolites and metabolic pathways across groups.

Notably, this study confirmed an inverse causal association between lithocholate sulfate and *Alistipes* via MR analysis, suggesting that microbial enrichment may be regulated by the host metabolic environment. As a secondary bile acid metabolized by gut microbiota, high levels of lithocholate sulfate inhibit microbial colonization by disrupting bacterial cell membranes (Zeng et al., 2021). Berberine intervention reduced lithocholate sulfate levels, potentially providing a suitable microenvironment for *Alistipes* enrichment. PDGFA, a member of the platelet-derived growth factor (PDGF) family, plays a key role in tissue repair by regulating extracellular matrix remodeling and cell proliferation (Zuo et al., 2023). Through MR and molecular docking, this study found that PDGFA can directly bind to lithocholate sulfate and reduce its levels. Functional validation experiments showed that inhibition of the PDGFA receptor (Lenvatinib) exacerbated the inflammatory response and colonic tissue damage in UC mice, and consequently led to a further deterioration of intestinal barrier function. Furthermore, the correlation analysis also confirmed that there was a highly significant negative correlation between the abundance of the genus *Alistipes* and lithocholate sulfate levels. The evidence for this direct association, together with the causal inference from the MR analysis, collectively supports that a decrease in lithocholate sulfate levels facilitates the enrichment of *Alistipes*.

Although previous studies have confirmed that berberine can regulate gut microbiota and affect bile acid metabolism, and that PDGFA plays a role in tissue repair (Lynch et al., 1987; Sun et al., 2017; Jael Teresa de Jesús et al., 2025), the causal links and directions between these findings remain unclear. The core advancement of this study lies in the first revelation of the “PDGFA-lithocholate sulfate-*Alistipes*” axis that spans the host and microbiota. Our findings elucidate a novel top-down pathway that is host gene-driven, metabolite-mediated, and ultimately precisely regulates specific gut microbiota, going beyond the traditional direct antibacterial or anti-inflammatory framework of berberine. This not only provides a new perspective for understanding its multi-target mechanism of action, but also connects host-driven tissue repair with microecological balance through specific metabolites, thereby revealing more refined potential regulatory targets for the treatment of UC. From the perspective of translational medicine, the discovery of this regulatory axis provides new potential targets for UC treatment: upregulating PDGFA expression or using lithocholate sulfate inhibitors may serve as strategies for combination therapy with berberine or alternative therapy. Meanwhile, monitoring lithocholate sulfate levels in serum or feces and *Alistipes* abundance may act as biomarkers for evaluating therapeutic efficacy, thereby promoting the development of precision medicine for UC.

However, this study still has some limitations. First, mouse UC models exhibit species differences from human diseases, which may limit the generalizability of the conclusions. Second, the validation of the “PDGFA-lithocholate sulfate-*Alistipes*” pathway is incomplete, and the upstream regulatory mechanism of PDGFA as well as the gut microbiota source of lithocholate sulfate have not been elucidated. Additionally, the 16S rRNA sequencing technology used in microbiome analysis has inherent limitations in functional gene interpretation. Finally, the study results have not been validated in human UC microbiome or metabolome datasets, which limits their clinical applicability to a certain extent. Therefore, future studies should validate the generalizability of this finding in human cohorts and integrate techniques such as metagenomics, transcriptomics, and gene editing to systematically clarify the molecular mechanism of this pathway.

## Conclusion

In summary, berberine significantly improved symptoms of ulcerative colitis in mice, regulated the gut microecology, and reshaped the composition of intestinal tissue metabolites. Using MR analysis, a unidirectional causal chain of PDGFA/lithocholate sulfate/*Alistipes* was first established. Animal experiments further verified that inhibition of the PDGFA receptor reversed the therapeutic effects of berberine and exacerbated inflammatory responses and intestinal mucosal barrier damage.

## Data Availability

The data presented in this study are deposited in the OMIX repository (https://ngdc.cncb.ac.cn/omix), accession number OMIX012548 (untargeted metabolomics data), and the European Nucleotide Archive (ENA) (https://www.ebi.ac.uk/ena), accession number PRJEB101457 (16S rRNA sequencing data).
