# Supplementary figures and images for: Regulatory effects of berberine on intestinal microecology in mice with ulcerative colitis
Source: Front Microbiol. 2025 Nov 12;16:1649947. doi: 10.3389/fmicb.2025.1649947 (PMC12647005; doi:10.3389/fmicb.2025.1649947)

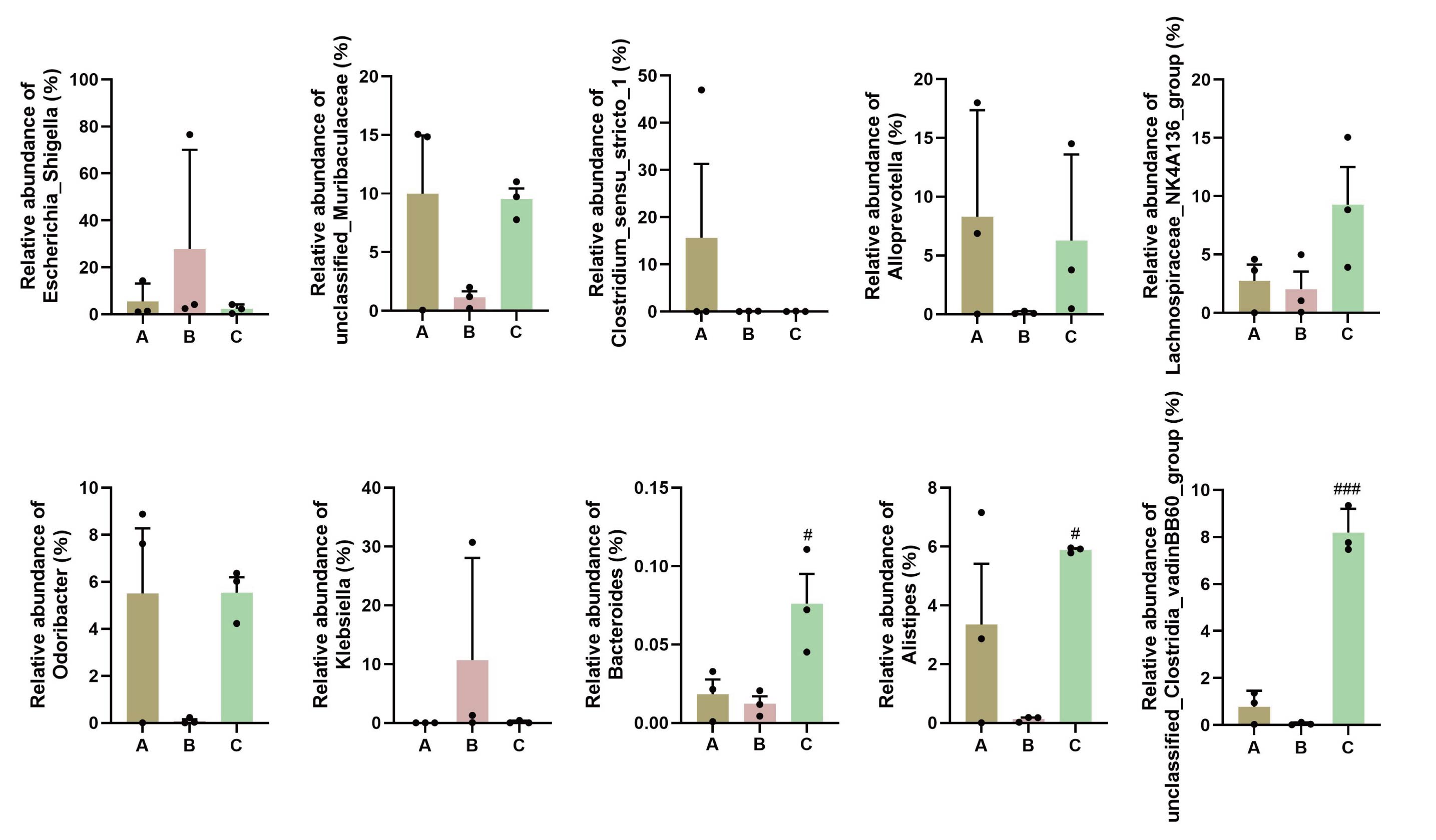

Supplement: Supplementary file 1 [file Image_1.JPEG]

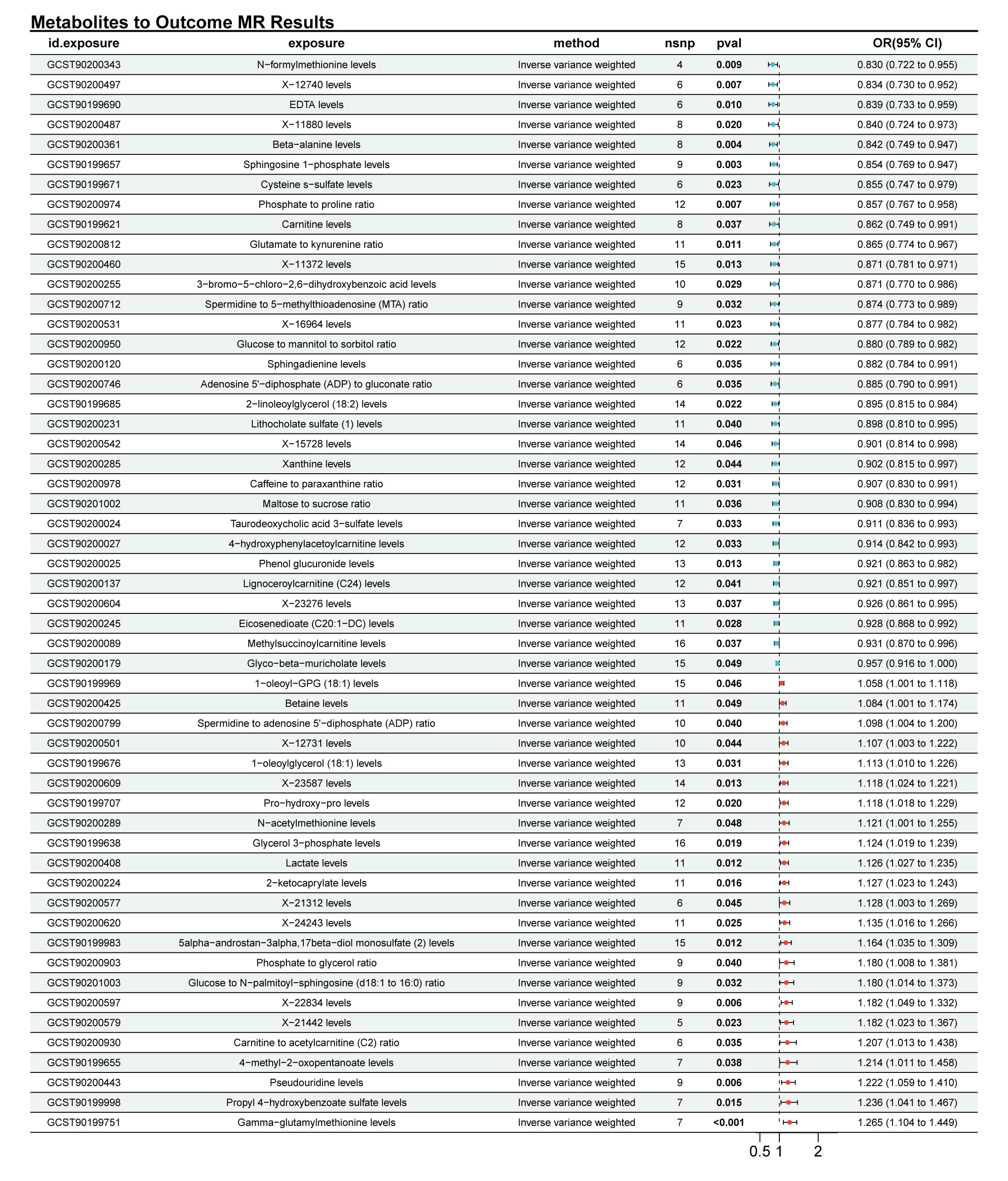

Supplement: Supplementary file 2 [file Image_2.JPEG]

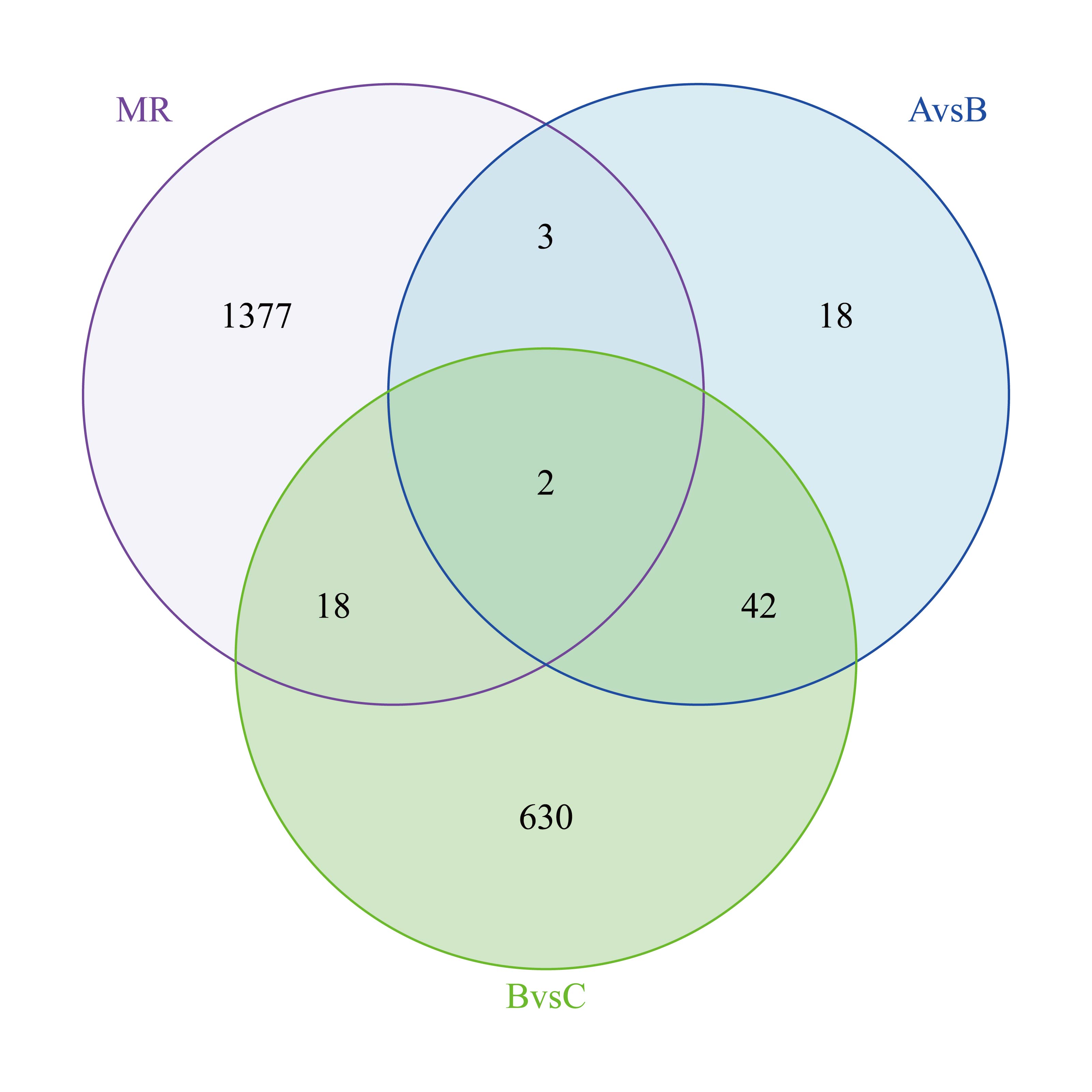

Supplement: Supplementary file 3 [file Image_3.JPEG]

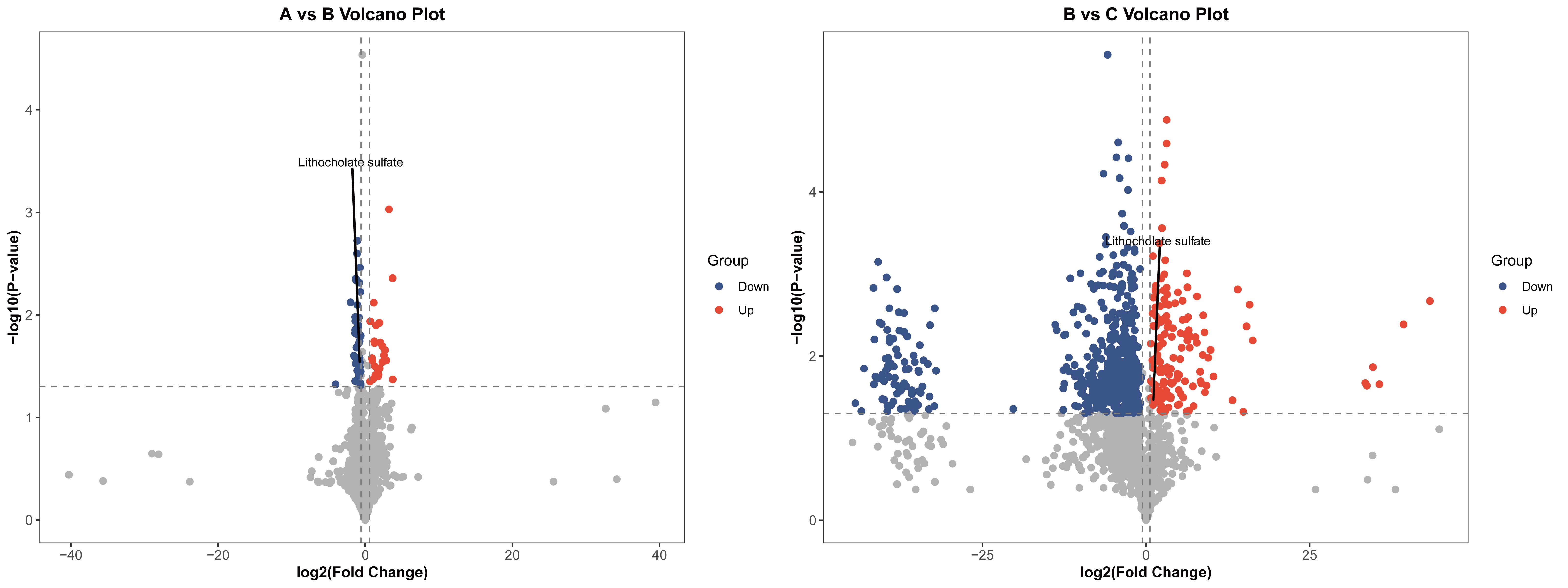

Supplement: Supplementary file 4 [file Image_4.JPEG]
